# Dr. Luke Arthur Harcourt

**Published:** 1916-05

**Authors:** 


**Dr. Luke Arthur Harcourt,** Buffalo 1868, of Sacramento, died in San Francisco, March 3, aged 77.

